# 400 AU/mL IgG protective threshold against SARS-CoV-2 XBB reinfection in Chinese inactivated vaccine recipients: implications for booster vaccination

**DOI:** 10.3389/fimmu.2026.1768679

**Published:** 2026-02-19

**Authors:** Zhiying Yin, Mengcheng Yin, Fei Zhao, Canya Fu, Wenjie Xu, Quanjun Fang, Xiaoying Gong, Shuangqing Wang, Canjie Zheng

**Affiliations:** 1 Department of Immunity, Quzhou Center for Disease Control and Prevention, Quzhou, Zhejiang, China; 2 Second Clinical Medical College, Tongji Medical College, Huazhong University of Science and Technology, Wuhan, Hubei, China; 3 School of Public Health, Zhejiang Chinese Medical University, Hangzhou, Zhejiang, China

**Keywords:** antibody dynamics, hybrid immunity, inactivated vaccine, protective threshold, SARS-CoV-2

## Abstract

**Background:**

Data on antibody dynamics and protective threshold generated by infection with Omicron subvariants of severe acute respiratory syndrome coronavirus 2 (SARS-CoV-2), particularly among populations that have primarily (>90%) received inactivated coronavirus disease 2019 (COVID-19) vaccines, remain limited. Using data from two large-scale paired serosurveys, we analyzed real-world changes in SARS-CoV-2 antibody levels associated with specific hybrid immunity.

**Methods:**

The history of COVID-19 vaccination and SARS-CoV-2 infection were recorded for each participant. Serum samples collected at three-month intervals were analyzed for antibody levels against nucleocapsid (N) and spike (S) proteins using chemiluminescent microparticle immunoassay (CMIA). Linear mixed-effects model (LMM) and restricted cubic spline analysis were applied to assess antibody dynamics and determine protection thresholds, respectively.

**Results:**

A total of 4,065 participants were recruited in February and May 2023, with 2,894 completing both sampling rounds. Over three months, median IgG antibody levels declined by 28% (from 396.39 to 285.80 AU/mL). Prior infection and vaccination were significantly associated with higher antibody levels, while increasing age correlated with an annual decay of 0.38 AU/mL (most prominent in adults ≥60 years, 32.7% of whom fell below the protective threshold by follow-up). The IgG threshold for protection against XBB reinfection was 400 AU/mL, and each additional vaccination reduced reinfection risk by 13–15%.

**Conclusion:**

The 400 AU/mL IgG threshold provides a actionable quantitative guideline for prioritizing booster doses in Chinese adults aged ≥60 years (the fastest antibody decay subgroup) and high-risk groups. Routine antibody testing combined with this threshold could optimize targeted COVID-19 vaccination strategies in inactivated vaccine-predominant populations.

## Introduction

Since its initial outbreak in late 2019, Severe Acute Respiratory Syndrome Coronavirus 2 (SARS-CoV-2) has transitioned into an endemic phase characterized by recurring waves of new variants, continuously challenging global public health systems (1). When faced with emerging infectious diseases, rapid development and deployment of vaccines are crucial for establishing herd immunity, which not only protects individuals but also reduces the spread of the disease within the population. This approach has received broad support from countries worldwide in combating SARS-CoV-2. While numerous countries have implemented massive coronavirus disease 2019 (COVID-19) vaccination campaigns, the primary vaccine platforms used vary significantly across regions. For instance, mRNA and adenovirus-vectored vaccines dominated the rollout in North America and Europe, whereas inactivated vaccines were extensively deployed in China and many countries in South America (e.g., Chile, Brazil) and the Middle East. Mass vaccination campaigns were launched in China, with inactivated vaccines accounting for over 90% of the administered doses (2). The country experienced its first major Omicron wave (predominantly BA.5/BF.7 lineages) between December 2022 and January 2023, followed by the emergence of the XBB variant in May 2023. This indicates that hybrid immunity (infection with SARS-CoV-2 and/or vaccination against COVID-19) within Chinese population is unique compared to other countries. However, robust data on the dynamics of specific hybrid immunity and correlates of protection, particularly against emerging Omicron subvariants, remain scarce.

Hybrid Immunity to SARS-CoV-2 is primarily mediated by humoral immune responses. Neutralizing antibodies (NAbs) are considered a gold standard correlate of protection because they directly inhibit viral entry into host cells by blocking the interaction between the spike protein and the angiotensin converting enzyme 2 (ACE2) receptor (3). However, standardized NAb assays are complex, time-consuming, and require specialized facilities (e.g., biosafety level 3 laboratories for live-virus neutralization tests) or advanced pseudovirus systems, which render them impractical for large-scale applications. Validation studies demonstrated that IgG titers exhibit a strong positive correlation with NAb levels (r > 0.85), and can be efficiently measured using standardized assays (4, 5). Therefore, IgG antibodies serve as an essential tool for monitoring infection rates and tracking epidemic progression (6).

The continuous evolution of Omicron subvariants, which exhibit increased immune escape capabilities, has significantly shortened the duration of protective immunity (7). In China, specific hybrid immunity against XBB variant remains unknown, and protective IgG threshold has not been validated. This has led to considerable uncertainty regarding the optimal timing and efficacy of booster doses (8). Recent evidence suggested that vaccination had a greater effect on IgG antibody levels than natural infection (9). However, the impact of demographic (e.g., age) and immunological factors (e.g., vaccination and/or infection) on antibody kinetics has not yet been comprehensively quantified. Given that repeated immune exposures lead to cumulative, dose-dependent enhancements in antibody levels (10). This study aims to further quantify the impact of age, vaccination, infection on short-term antibody dynamics, and to establish protection threshold of special hybrid Immunity in China against XBB variant through advanced statistical models, thereby providing evidence for timely and targeted vaccination strategies.

## Methods

### Study design and participants

**Figure 1:**
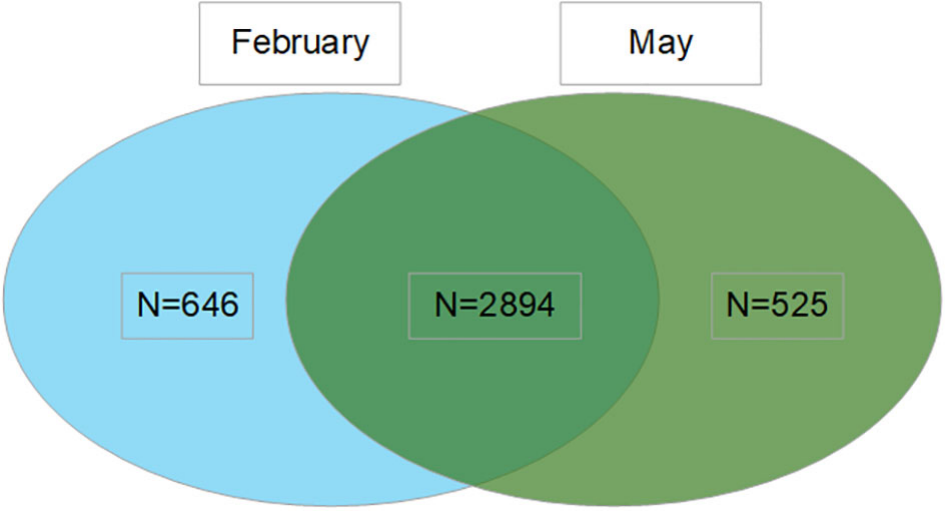
Flow of study participants through the two serial serosurveys. This Venn diagram illustrates the participant distribution from the two cross-sectional serosurveys conducted in February and May 2023. The blue circle represents the February survey (n=3540), the light green circle represents the May survey (n=3419), and the dark green overlap represents the participants (n=2894) included in both surveys, who constituted the longitudinal cohort for analysis.

A multi-stage stratified random sampling method was adopted for this study. First, stratification was performed at the county level, followed by the selection of urban or rural areas. The population was subsequently categorized into seven age groups: 3–9, 10–17, 18–29, 30–39, 40–59, 60–79, and 80 years or older. For the COVID-19 sentinel surveillance in January 2023, each age group was required to include at least 40 eligible individuals based on estimated infection rates. The initial survey in February 2023 included a total of 3,540 participants. In May 2023, the same cohort was followed up to collect data on vaccination status and infections occurring after the first sampling. Participants lost to follow-up were replaced with sex- and age-matched individuals, all of whom had completed the first-round questionnaire. Ultimately, 3,419 subjects participated in the May survey, and 2,894 individuals completed both rounds (**Figure 1**). Our cohort’s prior infection and vaccination coverage were consistent with the data of Zhejiang Province, supporting generalizability to Chinese inactivated vaccine recipients (**Supplementary Table S1**).

Each participant was required to complete face-to-face interview by trained personnel and to provide blood sample. A structured questionnaire covering demographic information, detailed COVID-19 vaccination history (dates and types of vaccines), and any prior laboratory-confirmed SARS-CoV-2 infections. To ensure accuracy, vaccination records were rigorously validated against the official “Zhejiang Immunization Program Smart Service Information System.” This system archives individualized data (including vaccine brand, manufacturer, dosage, lot number, expiration date, and administration time) linked to national identification cards via electronic supervision codes. SARS-CoV-2 infection was defined as a previous positive result via nucleic acid amplification test (NAAT) or rapid antigen test (RAT). Eligible participants were at least 3 years old and included non-local residents who had resided in the area for more than six months. Individuals with an active SARS-CoV-2 infection at the time of sampling were excluded. Written informed consent was obtained from all adult participants and from legal guardians of minors under age 18. The study protocol received approval from the Ethics Committee of the Quzhou Center for Disease Control and Prevention (Approval No. IRB-2023-R-001).

### Vaccination status classification

In accordance with China’s national COVID-19 vaccination guidelines and the “Technical Recommendations for COVID-19 Vaccination (First Edition),” participants were classified into five mutually exclusive categories based on their vaccination history prior to the blood draw date. The classification criteria are summarized in **Table 1**.

**Table 1:** Classification of vaccination status according to China’s COVID-19 technical guidelines.

Vaccination status	Definition
No vaccination	No history of COVID-19 vaccination.
Partial vaccinated	Having received any of the following: One dose of Ad5 within 14 days; One or two doses of Vero, with the last dose within 14 days; One, two, or three doses of CHO, with the last dose within 14 days.
Primary vaccinated	Having received any of the following: One dose of Ad5, two doses of Vero, or three doses of CHO, with the last dose more than 14 days ago; Two doses of Ad5 within 7 days; Two doses of Vero plus one booster dose (Vero, Ad5, or CHO) within 7 days.
First Booster	Having received any of the following: Two doses of Ad5 with the last dose more than 7 days ago; Two doses of Vero plus one booster (Vero/Ad5/CHO) with the last dose more than 7 days ago; Three doses of Ad5 within 7 days; Two doses of Vero plus two booster doses (Vero/Ad5/CHO) within 7 days; Three doses of CHO plus one booster (Vero/Ad5/CHO) within 7 days.
Second booster	Having received any of the following: Three doses of Ad5 with the last dose more than 7 days ago; Two doses of Vero plus two booster doses (Vero/Ad5/CHO) with the last dose more than 7 days ago; Three doses of CHO plus one booster (Vero/Ad5/CHO) with the last dose more than 7 days ago.

Ad5, adenovirus vector COVID-19 vaccine; Vero, inactivated COVID-19 vaccine; CHO, recombinant subunit COVID-19 vaccine.

### Laboratory testing

Serum samples were tested for anti-SARS-CoV-2 immunoglobulin G (IgG) antibodies using a chemiluminescent microparticle immunoassay (CMIA) on the iFlash 3000 automated analyzer (Shenzhen Yahuilong Biotechnology Co., Ltd.) with the iFlash SARS-CoV-2 IgG kit (Cat. No. C86095G). This assay uses magnetic beads coated with a combination of recombinant nucleocapsid (N) and spike (S) proteins to improve detection sensitivity. According to the manufacturer, the assay demonstrates 90% sensitivity and 95% specificity. IgG concentrations were quantified in arbitrary units per milliliter (AU/mL), derived from a calibration curve based on relative luminescence units (RLU). A cut-off value of 10 AU/mL was established based on receiver operating characteristic (ROC) curve analysis (AUC > 0.9), with values ≥10 AU/mL classified as seropositive and those below as seronegative.

To enhance the comparability of our IgG threshold with international studies, we referenced the established correlation between CMIA-detected anti-S (N+S) IgG and pseudovirus neutralization test (PVNT50) titers. Based on published data from Chinese populations receiving inactivated vaccines (4, 5, 9) and our own correlation analysis (r = 0.87, **Supplementary Table S2**) using a subset of samples (n=100) validated by a third-party laboratory (National Institute for Viral Disease Control and Prevention, China), we applied the conversion formula: PVNT50 = 0.31 × IgG (AU/mL)^0.42 (R² = 0.76) (5).

### Statistical analyses

All data were double-entered into EpiData 3.1 to ensure accuracy and subsequently analyzed using Microsoft Excel 2010, SPSS 27.0, and R software (v4.5.1). Anti-SARS-CoV-2 IgG levels were non-normally distributed and were summarized as median and interquartile range (IQR). Group comparisons employed the Wilcoxon rank-sum test for continuous variables and Chi-square test for categorical variables, as appropriate.

Linear mixed-effects model (LMM) was fitted with participant random intercepts to assess short-term antibody dynamics. Fixed effects included: (1) demographic factors: age (continuous, per year), sex (male/female); (2) immunological factors: vaccination status (five categories as defined in **Table 1**), prior infection status (uninfected/infected), reinfection status (unreinfected/reinfected); (3) temporal factors: survey timepoint (February/May); (4) potential confounders: comorbidity status (presence/absence of hypertension, diabetes, or chronic respiratory diseases), time since last infection (continuous, months), and time since last vaccination (continuous, months).

Time since last infection was calculated as the interval between the date of first positive NAAT/RAT and the first survey (February 2023). Time since last vaccination was the interval between the date of the most recent vaccine dose and the first survey. Model performance was evaluated using marginal R² (variance explained by fixed effects) and conditional R² (variance explained by both fixed and random effects). Multicollinearity among independent variables was assessed using variance inflation factors (VIF), all VIF values were below 2.0, indicating no concerning multicollinearity. To model the nonlinear relationship between baseline IgG levels and reinfection risk, restricted cubic spline analysis was performed with four knots placed at the 5th, 35th, 65th, and 95th percentiles of the IgG distribution. The reference value (OR = 1) was set at the 35th percentile. All analyses used a two-sided significance threshold of p < 0.05.

## Results

### Antibody levels between two surveys

**Figure 2:**
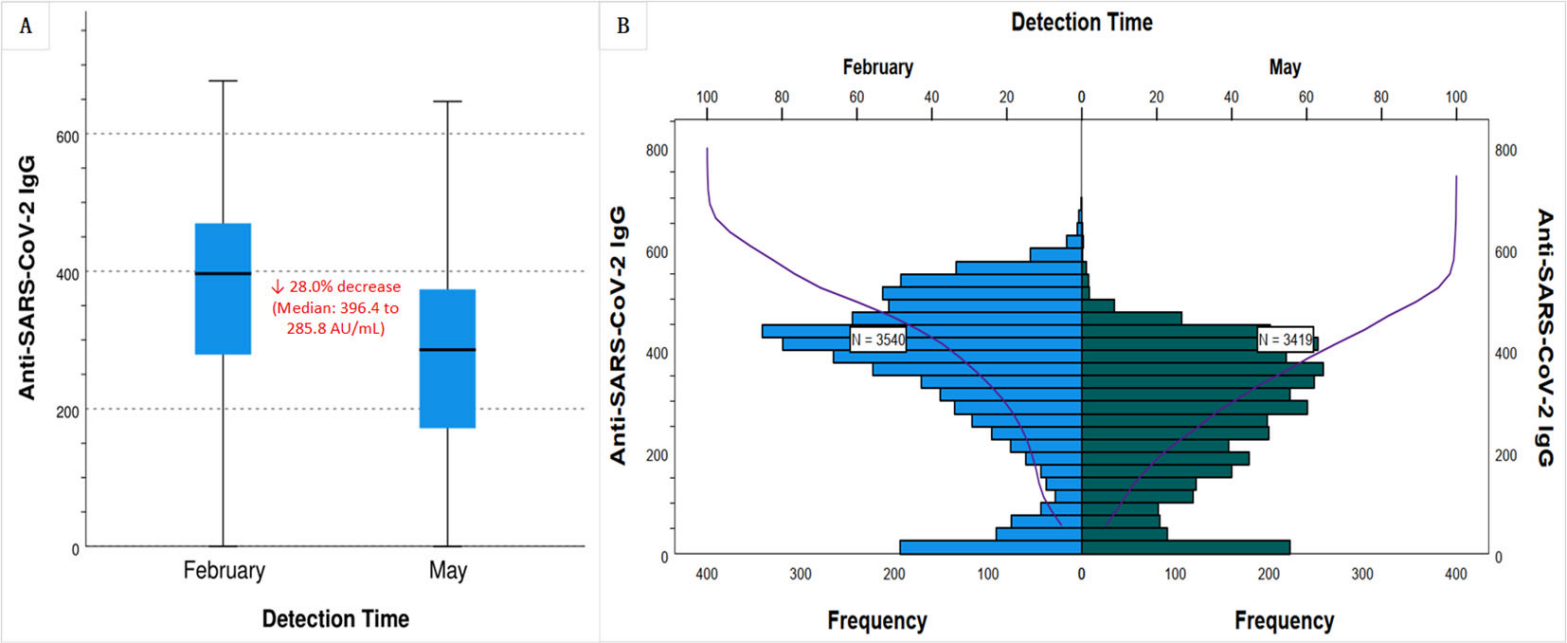
Waning of anti-SARS-CoV-2 IgG levels over a three-month period in Chinese population with inactivated vaccine-predominant hybrid immunity. Serum anti-SARS-CoV-2 IgG levels were measured between February and May 2023 in Quzhou, China. **(A)** Box plots show a 28.0% decline in the median IgG concentration, from 396.4 AU/mL in February 2023 to 285.8 AU/mL in May 2023. **(B)** Frequency distribution histograms (blue for February, green for May) and cumulative distribution curves (purple) demonstrate a leftward shift and flattened profile by May, indicating a population-wide decrease in antibody levels and increased interindividual variability.

The median anti-SARS-CoV-2 IgG concentration decreased by 28% (**Figure 2A**), from 396.39 AU/mL in February to 285.80 AU/mL in May 2023 (p < 0.001). The decay is visually confirmed in **Figure 2B**, which shows a pronounced leftward shift and flattening of the antibody distribution curve in May compared to February. The change indicates not only a widespread drop in antibody levels but also an increase in inter-individual variability. The baseline characteristics (e.g., sex, age distribution) of the participants in the two surveys were no significant differences. Both the February and May surveys revealed that age was inversely proportional to IgG levels, while vaccination status and infection frequency were directly proportional to IgG levels (**Table 2**).

**Table 2:** Compare anti-SARS-CoV-2 IgG levels in two cross-sectional serum surveys in February and May, 2023.

Characteristics	February			May			*U/χ^2^*	*p*
	N (%)	Median (IQR)	*p*	N (%)	Median (IQR)	*p*		
All	3540 (100.00)	396.39 (278.74, 469.69)		3419 (100.00)	285.80 (171.65, 373.69)		634.52	<0.001
Gender			0.483			0.084	0.587	0.444
Male	1802 (50.90)	394.52 (278.15, 464.71)		1709 (49.98)	279.43 (166.84, 373.77)			
Female	1738 (49.09)	397.06 (278.85, 472.14)		1710 (50.01)	290.84 (176.47, 373.80)			
Age (year)			<0.001			<0.001	0.191	0.909
3~17	1017 (28.73)	415.74 (302.75, 487.22)		996 (29.13)	320.00 (219.22, 399.12)			
18~59	1535 (43.36)	391.66 (288.23, 461.75)		1482 (43.35)	273.06 (172.98, 360.77)			
60+	988 (27.91)	381.45 (230.49, 464.06)		941 (27.52)	259.34 (128.07, 361.91)			
Vaccination status			<0.001			<0.001	30.724	<0.001
No vaccination	163 (4.60)	46.90 (20.75, 92.89)		84 (2.46)	77.88 (15.56, 310.26)			
Partial vaccinated	61 (1.72)	127.35 (39.03,407.99)		42 (1.23)	206.46 (50.22, 340.46)			
Primary vaccinated	1115 (31.50)	412.85 (310.12, 480.86)	1176 (34.40)	310.16 (199.41, 394.71)				
First booster	1640 (46.33)	380.19 (273.72, 458.14)		1561 (45.66)	253.78 (152.03, 340.99)			
Second booster	561 (15.85)	430.96 (372.52, 503.70)		556 (16.26)	337.24 (242.59, 401.94)			
Infection frequency			<0.001			<0.001	281.07	<0.001
Uninfected	622 (17.57)	70.11 (17.10, 183.83)		470 (13.75)	90.15 (18.77, 233.59)			
One time	2901 (81.95)	419.26 (350.57, 488.88)		2651 (77.54)	288.59 (192.95, 368.85)			
Two times	17 (0.48)	438.18 (389.62, 481.32)		298 (8.72)	409.18 (332.23, 445.94)			

Data are presented as number (percentage) for categorical data, median (IQR) for nonparametrically distributed data. Wilcoxon rank sum test were performed for comparison of the continuous variables according to the data distribution, and Chi-square test for the categorical variables.

### Short-term antibody dynamics

**Figure 3:**
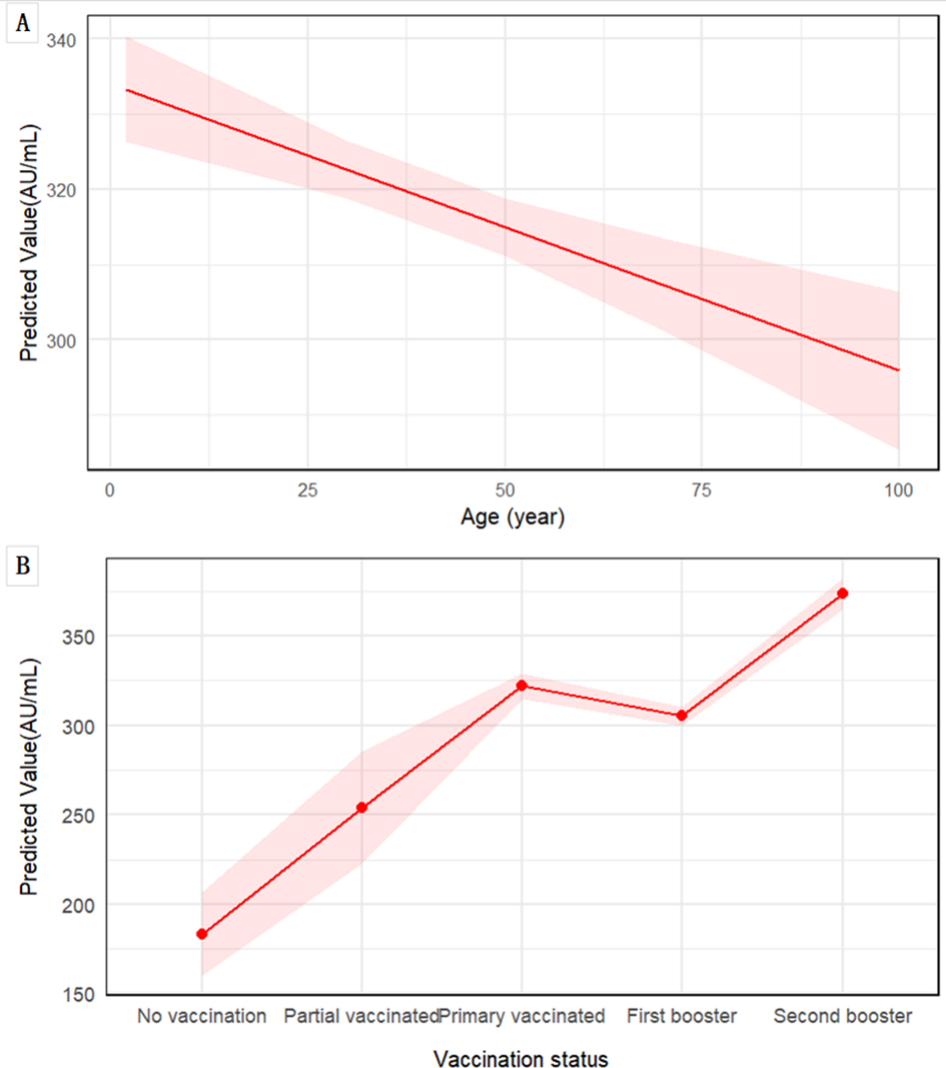
Associations of age and vaccination status with predicted anti-SARS-CoV-2 IgG levels. Predictions are derived from LMM, adjusted for sex, prior SARS-CoV-2 infection status, and testing time. **(A)** The marginal effect of age on IgG level. The solid red line represents the predicted values from the model, with the shaded area indicating the 95% CI. **(B)** Estimated marginal means of IgG levels by vaccination status, and shaded area represent the 95% CI.

A total of 2,894 participants underwent serial serological testing in February and May, with no COVID-19 vaccine boosters administered between the two surveys. Among the 2,827 vaccinated individuals, a total of 7,965 doses of COVID-19 vaccines were administered. The vast majority of doses were inactivated vaccines (Vero cell, 91%), with recombinant subunit (CHO, 6.03%) and adenovirus vector (Ad5, 2.97%) vaccines constituting a small fraction of the total (**Supplementary Table S3**). Consequently, the statistical power was insufficient to conduct a robust comparative analysis of antibody dynamics or protective effects across different vaccine platforms. According to infection status of individuals during the two surveys, they were divided into unreinfected and reinfection groups. LMM was fitted with IgG levels as the dependent variable, incorporating fixed effects for group, gender, age, vaccination status, prior infection, and test time, along with random intercepts for each individual. The model exhibited substantial explanatory power, with conditional and marginal R² values of 0.619 and 0.402, respectively. The intercept IgG level was 89.82 AU/mL. Significant predictors of IgG levels included reinfection status (+23.10 AU/mL), prior infection (+188.10 AU/mL), and testing in May (–100.85 AU/mL compared to February). No sex-based differences were observed. LMM revealed that increasing age was independently and significantly associated with lower anti-SARS-CoV-2 IgG levels (β = -0.38 AU/mL per year, 95% CI: -0.55 to -0.22, p < 0.001) (**Table 3**). This negative association was visually presented in **Figure 3A**, which shows the predicted decline in IgG levels across the age spectrum. In contrast, a clear positive association was observed between vaccination status and antibody levels, with individuals receiving a second booster demonstrating the highest predicted IgG levels (**Figure 3B**).

**Table 3:** Short-term anti-SARS-CoV-2 IgG dynamics from linear mixed-effects modeling in a paired serological cohort.

Variable	NO. of subjects	Estimate (β)	Std. error	95%CI	*p*-value	Adjusted estimate (β)*	Adjusted 95% CI	Adjusted p-value
Fixed effects								
Intercept		89.81	12.80	(64.77, 114.86)	<0.001	92.35	(66.91, 117.79)	<0.001
Group								
Unreinfected (Ref.)	2454							
Reinfection	440	23.10	5.06	(13.20, 33.00)	<0.001	22.78	(12.95, 32.61)	<0.001
Gender								
Male (Ref.)	1433							
Female	1461	-0.31	3.57	(-7.30, 6.67)	0.930	-0.52	(-7.58, 6.54)	0.897
**Age (per year)**	2894	-0.38	0.08	(-0.55, -0.22)	<0.001	-0.39	(-0.56, -0.23)	<0.001
Vaccination status								
No vaccination (Ref.)	67							
Partial vaccinated	36	70.71	19.78	(32.00, 109.42)	<0.001	71.24	(32.31, 110.17)	<0.001
Primary vaccinated	972	138.72	12.29	(114.68, 162.77)	<0.001	136.89	(112.75, 161.03)	<0.001
First booster	1324	121.82	12.16	(98.02, 145.62)	<0.001	120.35	(96.48, 144.22)	<0.001
Second booster	495	190.13	12.64	(165.40, 214.87)	<0.001	188.57	(163.69, 213.45)	<0.001
Prior infection								
Uninfected (Ref.)	500							
Infection	2394	188.10	4.83	(178.65, 197.55)	<0.001	185.72	(176.18, 195.26)	<0.001
Test time								
February (Ref.)	2894							
May	2894	-100.85	2.43	(-105.61, -96.09)	<0.001	-101.23	(-106.02, -96.44)	<0.001

β, Regression coefficient; CI, Confidence Interval; AIC, Akaike Information Criterion; R2, Coefficient of Determination.

*Adjusted for time since last infection and time since last vaccination.

Model fit statistics: AIC = 70,990.05; Conditional R² = 0.619; Marginal R² = 0.402;

### Protective thresholds against reinfection

A cohort of 2,894 individuals was stratified by infection status from February to May into non-reinfected and reinfected groups. Age and baseline IgG levels (measured in February) were categorized into quartiles based on their distributions within the non-reinfected group. Binary logistic regression analysis was conducted with reinfection as the outcome variable and gender, age group, vaccination status, prior infection history, and baseline IgG level as independent predictors. Univariate analyses showed no significant association between reinfection risk and age quartiles; however, vaccination status, prior infection history, and baseline IgG quartiles were significantly associated with reinfection (all p < 0.05). All VIF values ranged from 1.00 to 1.39, indicating absence of substantial multicollinearity. In the multivariate model adjusted for potential confounders, prior infection history was not independently associated with reinfection risk. Nevertheless, statistically significant dose–response relationships were observed between reinfection risk and both vaccination status (p = 0.02) and baseline IgG levels (p < 0.001), as summarized in **Table 4**.

**Table 4:** Odds ratios (95% CIs) for reinfection based on binary logistic regression.

Variables	N (non-reinfected/reinfected)	Univariate model		Multivariate model	
		OR (95%CI)	*p*-value	OR (95%CI)	*p*-value
Gender			0.048		0.048
Male	1258/203	1.00 (reference)		1.00 (reference)	
Female	1196/237	1.23 (1.00, 1.51)		1.25 (1.00, 1.55)	
Age (years), quartiles			0.852^a^		0.716^a^
Q1 (3.0 - 14.1)	617/105	1.00 (reference)	0.833	1.00 (reference)	0.669
Q2 (14.1 - 34.4)	609/117	1.13 (0.85, 1.50)	0.406	1.14 (0.79, 1.65)	0.475
Q3 (34.4 - 62.0)	614/106	1.01 (0.76, 1.36)	0.923	0.96 (0.62, 1.47)	0.845
Q4 (≥ 62.0)	614/112	1.07 (0.80, 1.43)	0.638	0.98 (0.66, 1.47)	0.938
Vaccination status			0.017^a^		0.020^a^
No vaccination	42/25	1.00 (reference)	<0.001	1.00 (reference)	0.002
Partial vaccinated	27/9	0.56 (0.23, 1.38)	0.208	0.91 (0.35, 2.37)	0.846
Primary vaccinated	829/143	0.29(0.17, 0.49)	<0.001	0.68 (0.39, 1.19)	0.180
First booster	1140/184	0.27 (0.16, 0.46)	<0.001	0.51 (0.29, 0.90)	0.021
Second booster	416/79	0.32(0.18, 0.55)	<0.001	0.90 (0.49, 1.66)	0.739
Linear model^b^		0.85 (0.76, 0.96)		0.87 (0.77, 0.98)	
Prior infection			<0.001		0.110
Uninfected	349/151	1.00 (reference)		1.00 (reference)	
Infection	2105/289	0.32 (0.25, 0.40)		0.80 (0.61, 1.05)	
IgG (February) (AU/mL)			<0.001^a^		<0.001^a^
Q1 (< 324.0)	613/265	1.00 (reference)	<0.001	1.00 (reference)	<0.001
Q2 (324.0 – 415.1)	615/99	0.37 (0.29, 0.48)	<0.001	0.41 (0.30, 0.55)	<0.001
Q3 (415.1 – 493.6)	613/62	0.23 (0.17, 0.32)	<0.001	0.24 (0.17, 0.34)	<0.001
Q4 (≥ 493.6)	613/14	0.05 (0.03, 0.09)	<0.001	0.05 (0.03, 0.10)	<0.001
Linear model^b^		0.99 (0.99, 0.99)		0.99 (0.99, 0.99)	

*^a^p*-value for trend. *^b^*The risk of reinfection decreased with each unit increase in the median IgG level across quartiles and with each incremental level of vaccination.

**Figure 4:**
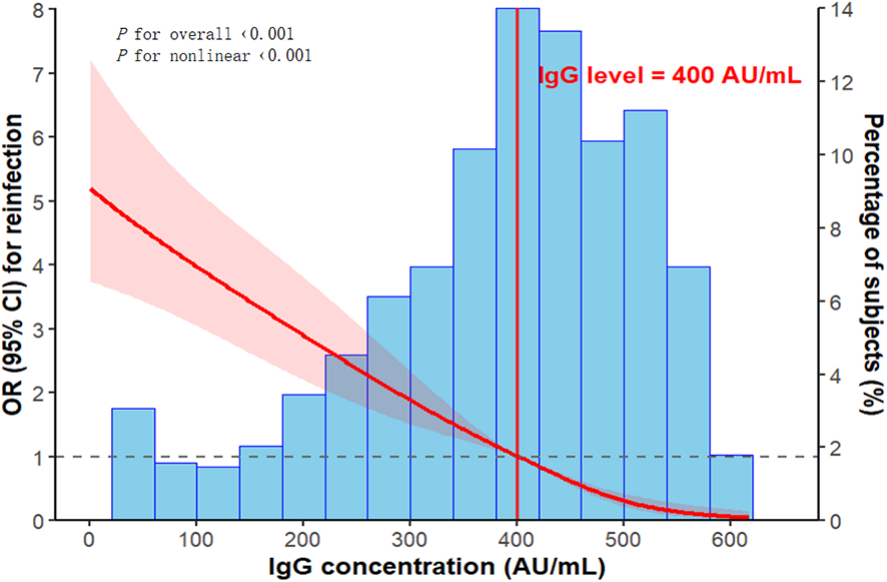
Non-linear association between baseline IgG levels and the risk of reinfection, analyzed using restricted cubic splines with adjustment for potential confounders. The red line represents the adjusted OR, with the shaded region denoting the 95% CI. The vertical red line marks the identified protective threshold of 400 AU/mL. The reference category corresponds to the 35th percentile of baseline IgG levels (324.0 AU/mL). The histogram illustrates the distribution of baseline IgG levels.

Each incremental level in vaccination status was associated with a 13–15% reduction in the risk of reinfection. Furthermore, for each unit increase in the median IgG level across quartiles, reinfection risk decreased significantly by approximately 0.7%. A dose-response relationship between baseline IgG levels and reinfection risk was further evaluated using restricted cubic splines, revealing a non-linear, inverse J-shaped association with a critical threshold identified at 400 AU/mL (**Figure 4**). Below this threshold, reinfection risk increased sharply; above it, the risk declined and eventually plateaued.

## Discussion

The 28% decline in median IgG levels over three months observed in our cohort was notably slower than the more rapid waning (often 30-40% over similar periods) frequently reported in mRNA-vaccinated populations (11). This observation is consistent with broader evidence comparing vaccine platforms. For instance, the large multi-center ORCHESTRA project, which followed mRNA-vaccinated European participants, demonstrated significant antibody titer decay after both primary and booster vaccination courses, highlighting a characteristic kinetic pattern of strong initial response followed by swift decline (12, 13). A systematic review by Rahmani et al. further synthesized evidence that antibody correlates of protection in mRNA vaccine recipients may require higher peak titers or more frequent boosting due to this waning profile (14). This attenuated decay observed in our cohort may be linked to the unique immune background of inactivated vaccines, which present a broader array of antigens beyond the spike protein alone, potentially leading to a more sustained, if less peaky, immune stimulation (15, 16). This distinction underscores the critical need for population-specific protection thresholds, as timing booster doses based on mRNA vaccine data may not be optimal for inactivated vaccine recipients. Supporting this, longitudinal studies like that by Fujiya et al. have emphasized the importance of tailoring booster strategies based on vaccine-specific antibody kinetics (17).

LMM analysis revealed that both prior infection (+188 AU/mL) and receipt of a second booster dose (+190 AU/mL) were associated with comparable increases in antibody levels. This striking similarity in the magnitude of the response highlights a robust ‘hybrid immunity’ effect, wherein the combination of vaccination and natural infection engenders a broad and durable immunological memory (18, 19). These findings provide mechanistic insights into the cumulative effect of immune imprinting: successive antigenic exposures—whether from vaccination or infection—preferentially activate and refine pre-existing memory B cell clones. This process results in elevated production of cross-reactive antibodies and may promote the development of longer-lived plasma cells, thereby strengthening and prolonging the humoral immune response (20–22). Notably, adults ≥60 years exhibited the lowest IgG levels and fastest decay (0.38 AU/mL/year), with over one-third failing to maintain the 400 AU/mL threshold at 3 months. This highlights the need for prioritized booster doses in this subgroup, as supported by previous studies linking immunosenescence to reduced vaccine responsiveness (23–25).

Our protective IgG threshold of 400 AU/mL translates to a PVNT50 titer of ~1:86 (95% CI: 1:73–1:101), which is consistent with the PVNT50 threshold of 1:80–1:100 reported for Omicron subvariants in hybrid immunity populations (26, 27). The distinct immune kinetics elicited by different vaccine platforms, as discussed above, are directly reflected in these differing correlates of protection.​ Our threshold is substantially lower than the 6321 BAU/mL reported for mRNA-vaccinated cohorts (27). This discrepancy not only underscores the importance of population-specific thresholds (15, 28) but also suggests that the broader antigenic repertoire of inactivated vaccines may lead to a qualitatively different antibody repertoire that is functional at a lower titer, despite higher titers being required for protection with spike-focused vaccines. The circulating variant during the study period (XBB) exhibits extensive immune escape properties, which likely increases the threshold of neutralizing antibodies required for protection compared to earlier variants (29). However, our threshold substantially exceeds the 264 BAU/mL required for 50% protection against ancestral strains (30), consistent with the established understanding that immune-evasive variants such as Omicron necessitate higher levels of humoral immunity to confer protection (31).

Our cohort exhibits slight deviations from national and Zhejiang Provincial demographic data (e.g., higher proportion of children aged 3–17 years; **Supplementary Table S1**), which were intentional to ensure sufficient statistical power for age-stratified analyses, an understudied subgroup in COVID-19 serological research. Despite these deviations, the core characteristics of our cohort (≥90% inactivated vaccine coverage, infection rate consistent with regional data, and age-related antibody decay pattern) are consistent with the broader Chinese population receiving inactivated vaccines. Therefore, our findings (400 AU/mL protective threshold, prioritization of boosters for adults ≥60 years) are generalizable to mainland China’s inactivated vaccine-predominant populations, particularly in regions with inactivated vaccine-predominant vaccination strategies.

The study has several key methodological strengths that enhance the validity of our findings. First, the large-scale, paired serosurvey design with a high participant retention rate provides robust longitudinal data on antibody dynamics, minimizing selection bias. Second, the application of advanced statistical approaches, including linear mixed-effects models to account for repeated measures and restricted cubic splines to identify a non-linear threshold, offers a sophisticated analysis beyond simple descriptive statistics. Furthermore, vaccination history was objectively validated against a provincial-level immunization information system, ensuring accurate exposure classification. Ultimately, by establishing a clear protective threshold against XBB reinfection, this study addresses a critical evidence gap for the large global population primarily vaccinated with inactivated vaccines, for whom specific correlates of protection were previously lacking.

This study has several limitations. First, previous infection was defined by positive NAAT or RAT results. However, incomplete testing across the study period may have led to underdetection of asymptomatic infections and underestimation of the reinfection rate, slightly overestimating the protective threshold (400 AU/mL IgG). Future studies should quantify the asymptomatic infection proportion to refine this threshold. Second, as a respiratory pathogen, SARS-CoV-2 is first encountered at the mucosal surfaces. Mucosal antibodies, particularly secretory IgA, serve as the primary defense at the site of viral entry and play a critical role in achieving sterilizing immunity and reducing transmission (32, 33). A recognized limitation of our study is its exclusive focus on systemic IgG responses, without assessing mucosal immunity. Therefore, future studies should incorporate the measurement of mucosal antibody levels (e.g., in saliva or nasal secretions) to provide a more comprehensive evaluation of immune protection against SARS-CoV-2 infection and transmission. Third, the follow-up period of this study was limited to three months. While this allows for the analysis of short-term antibody dynamics and the establishment of an initial protective threshold, it does not provide data on the long-term durability of antibody protection or the stability of the 400 AU/mL threshold against evolving variants. Therefore, longer-term longitudinal studies are needed to validate the durability of this threshold and its applicability against future SARS-CoV-2 variants. Finally, our study population was predominantly vaccinated with inactivated vaccines (91% of all doses), as detailed in **Supplementary Table S3**. While this reflects the actual vaccination landscape in China, it resulted in limited sample sizes for other vaccine platforms (e.g., recombinant subunit and adenovirus vector vaccines). This precluded a meaningful statistical comparison of immunogenicity across different vaccine types, which remains an important question for future research in more diversely vaccinated cohorts.

In conclusion, our study demonstrates that hybrid immunity, predominantly induced by inactivated COVID-19 vaccines in the Chinese population, is characterized by a 28% median decline in serum IgG levels over three months. Prior SARS-CoV-2 infection and vaccination—particularly receipt of a second booster dose—are the strongest predictors of elevated antibody concentrations, whereas advancing age is associated with a steady annual IgG decay of 0.38 AU/mL. Critically, we identified a protective threshold of 400 AU/mL for IgG against XBB variant reinfection, a finding with direct implications for targeted booster vaccination strategies. Given the continuous evolution of SARS-CoV-2 variants, this threshold highlights the necessity of dynamic monitoring and regular updates to immunity benchmarks, especially for high-risk groups such as adults aged ≥60 years who experience accelerated antibody waning. Integrating this quantitative criterion into routine antibody testing could optimize the timing and prioritization of booster doses, strengthening public health preparedness against emerging Omicron subvariants in inactivated vaccine-predominant populations.

## Data Availability

The original contributions presented in the study are included in the article/**Supplementary Material**. Further inquiries can be directed to the corresponding authors.
