# Supplementary material for: 400 AU/mL IgG protective threshold against SARS-CoV-2 XBB reinfection in Chinese inactivated vaccine recipients: implications for booster vaccination
Source: Front Immunol. 2026 Feb 19;17:1768679. doi: 10.3389/fimmu.2026.1768679 (PMC12960472; doi:10.3389/fimmu.2026.1768679)
Supplement: Supplementary file 1 [file Table1.pdf]

Supplementary Table S1. Comparison of cohort characteristics with national and Zhejiang Provincial population data.

Characteristic	Study Cohort (N=3540)	National Population (2020)	Zhejiang Provincial Population (2020)	Zhejiang COVID-19 Vaccination (Dec 2022)
Age group, n (%)				
3–17 years	1017 (28.73)	339.2 million (23.95%)	19.6 million (22.81%)	-
18–59 years	1535 (43.36)	829.6 million (58.79%)	51.0 million (59.12%)	-
≥60 years	988 (27.91)	240.9 million (17.26%)	15.6 million (18.07%)	-
Sex, n (%)				
Male	1802 (50.90)	723.3 million (51.24%)	43.8 million (50.98%)	-
Female	1738 (49.10)	688.5 million (48.76%)	42.2 million (49.02%)	-
Vaccination coverage, n (%)				
≥1 dose	3377 (95.40)	-	-	92.7% (≥3 years old)
≥2 doses	3124 (88.25)	-	-	89.8% (≥3 years old)
Prior infection, n (%)	2918 (82.43)	-	-	78–85% (Zhejiang, Dec 2022–Jan 2023)

Note: National Population Census data were retrieved from the National Bureau of Statistics of China (http://www.stats.gov.cn). Zhejiang Provincial population and vaccination data were retrieved from the Zhejiang Provincial Health Commission (http://wsjkw.zj.gov.cn).
