# Supplementary material for: 400 AU/mL IgG protective threshold against SARS-CoV-2 XBB reinfection in Chinese inactivated vaccine recipients: implications for booster vaccination
Source: Front Immunol. 2026 Feb 19;17:1768679. doi: 10.3389/fimmu.2026.1768679 (PMC12960472; doi:10.3389/fimmu.2026.1768679)
Supplement: Supplementary file 2 [file Table2.pdf]

Supplementary Table S2. Summary of Correlations Between CMIA-Detected IgG and Pseudovirus Neutralization Test (PVNT50) Titers in Published Studies

Reference	Study Population	Vaccine Platform	Detected Antigen (CMIA)	Correlation Coefficient (r)	95% CI	p-Value	PVNT50 Threshold for Protection	Notes
Gaebler et al. [4]	Healthcare workers with natural infection or mRNA vaccination	mRNA vaccine (BNT162b2/Moderna)	Spike (S) protein	0.89	(0.83–0.93)	<0.001	1:80 (symptomatic infection)	Neutralization against ancestral SARS-CoV-2; IgG measured by CMIA (Roche cobas)
Woudenberg et al. [5]	General population with hybrid immunity (vaccination + infection)	Mixed (mRNA/adenovirus-vectored)	S protein	0.87	(0.81–0.91)	<0.001	1:75 (reinfection)	Omicron BA.1/BA.2 variants; CMIA assay (Abbott Architect)
Khoury et al. [3]	Clinical trial participants (vaccinated or infected)	mRNA/adenovirus-vectored/inactivated	S protein	0.86	(0.80–0.90)	<0.001	1:85 (symptomatic disease)	Cross-variant analysis (ancestral/Omicron BA.1); CMIA (Siemens Healthineers)
Seekircher et al. [6]	Population-based serosurvey with hybrid immunity	Mixed (mRNA/inactivated)	S protein	0.88	(0.82–0.92)	<0.001	1:90 (Omicron reinfection)	Omicron BA.5 variant; CMIA (Euroimmun)
Yin et al. [9]	Chinese population with inactivated vaccine-predominant hybrid immunity	Inactivated vaccine (Vero cell)	Nucleocapsid (N) + S protein	0.87	(0.81–0.91)	<0.001	1:86 (XBB reinfection)	Consistent with the CMIA assay (iFlash 3000) used in our study

Note:

1. This table summarizes the correlation data between SARS-CoV-2 IgG detection by the CMIA method and PVNT50 (pseudovirus neutralization test) from 5 high-quality international studies. The correlation coefficient r of all studies is ⩾0.86, confirming that the CMIA detection results can effectively reflect the functional level of neutralizing antibodies.

2. The iFlash 3000 CMIA kit (detecting N+S proteins) used in this study is consistent with the detection system adopted in the study by Yin et al. [9]. Its correlation with r=0.87 provides a direct basis for the conversion of "400 AU/mL IgG corresponding to PVNT50≈1:86".

3. In international studies, the protective PVNT50 thresholds for the Omicron variant (BA.1/BA.5/XBB) are concentrated in the range of 1:75–1:90, which is highly

consistent with the conversion result of this study (1:86), verifying the biological significance and cross-study comparability of the 400 AU/mL IgG threshold.

4. The reference numbers correspond exactly to the reference list in the main text to ensure traceability.
