# Supplementary material for: 400 AU/mL IgG protective threshold against SARS-CoV-2 XBB reinfection in Chinese inactivated vaccine recipients: implications for booster vaccination
Source: Front Immunol. 2026 Feb 19;17:1768679. doi: 10.3389/fimmu.2026.1768679 (PMC12960472; doi:10.3389/fimmu.2026.1768679)
Supplement: Supplementary file 3 [file Table3.pdf]

Supplementary Table S3. Distribution of COVID-19 vaccine types and brands administered in the cohort.

Number of administered doses	Vaccine Platform			Total
	Vero	CHO	Ad5	
First dose	2771	42	16	2829
Second dose	2748	38	13	2799
Third dose	1636	187	45	1868
Fourth dose	93	213	163	469
Fifth dose	0	0	0	0
Total	7248	480	237	7965

Abbreviations: Ad5: adenovirus vector COVID-19 vaccine, Vero: inactivated COVID-19 vaccine, CHO: recombinant subunit COVID-19 vaccine
